# Peripheral Nerve Ligation Elicits Widespread Alterations in Cortical Sensory Evoked and Spontaneous Activity

**DOI:** 10.1038/s41598-019-51811-8

**Published:** 2019-10-25

**Authors:** Donovan M. Ashby, Jeffrey LeDue, Timothy H. Murphy, Alexander McGirr

**Affiliations:** 1 Hotchkiss Brain Institute, University of Calgary, Calgary, Canada; 2 Department of Psychiatry, Cumming School of Medicine, University of Calgary, Calgary, Canada; 3 Mathison Centre for Mental Health Research and Education, University of Calgary, Calgary, Canada; 4 Djavad Mowafaghian Centre for Brain Health, University of British Columbia, Vancouver, Canada; 5 Department of Psychiatry, University of British Columbia, Vancouver, Canada

**Keywords:** Chronic pain, Cortex

## Abstract

Peripheral neuropathies result in adaptation in primary sensory and other regions of cortex, and provide a framework for understanding the localized and widespread adaptations that arise from altered sensation. Mesoscale cortical imaging achieves high temporal resolution of activity using optical sensors of neuronal activity to simultaneously image across a wide expanse of cortex and capture this adaptation using sensory-evoked and spontaneous cortical activity. Saphenous nerve ligation in mouse is an animal model of peripheral neuropathy that produces hyperalgesia circumscribed to the hindlimb. We performed saphenous nerve ligation or sham, followed by mesoscale cortical imaging using voltage sensitive dye (VSD) after ten days. We utilized subcutaneous electrical stimulation at multiple stimulus intensities to characterize sensory responses after ligation or sham, and acquired spontaneous activity to characterize functional connectivity and large scale cortical network reorganization. Relative to sham animals, the primary sensory-evoked response to hindlimb stimulation in ligated animals was unaffected in magnitude at all stimulus intensities. However, we observed a diminished propagating wave of cortical activity at lower stimulus intensities in ligated animals after hindlimb, but not forelimb, sensory stimulation. We simultaneously observed a widespread decrease in cortical functional connectivity, where midline association regions appeared most affected. These results are consistent with localized and broad alterations in intracortical connections in response to a peripheral insult, with implications for novel circuit level understanding and intervention for peripheral neuropathies and other conditions affecting sensation.

## Introduction

Peripheral neuropathies can result in hyperalgesia and allodynia, and are thus a major cause of chronic pain^1^. These localized peripheral insults cause a range of peripheral and central nervous system adaptations that ultimately result in significant direct and indirect health costs^2^.

Peripheral neuropathies cause alterations to sensory circuits inducing adaptations in peripheral nociceptive fibres, descending control system, and excitatory transmission in the spinal cord dorsal horn^3–7^. Recently, reorganization of higher order sensory and affective processing regions has been observed, leading to a focus on understanding changes occurring in higher CNS regions and how these alterations might contribute to the persistence of altered nociception and its broader repercussions^8–10^.

Cortical sensory processing in the absence of pathology involves thalamocortical inputs to primary somatosensory cortex, and from this primary sensory-evoked response propagates a wave of activity that spreads via horizontal connections beyond the primary response region^11,12^. Though the function of these waves is unclear, several roles have been proposed including temporal information relating to stimulus onset and multisensory integration^12^. These long-range intracortical connections indicate recurrent interactions within cortex^13^, and we hypothesized that they would be susceptible to pathological adaptations and associated with changes in spontaneous activity dynamics in which they participate.

Experimental chronic peripheral neuropathy^14–18^ perturbs sensory circuits by selectively inducing aberrant sensory information to a localized region of somatosensory cortex and produces a behavioral phenotype of hyperalgesia similar to clinical neuropathic pain. Synaptic changes occur rapidly in rodent models of neuropathy^19,20^, with stabilization and sustained changes reflected in large scale brain network changes^21^. Using the saphenous nerve ligation model together with millisecond resolution voltage sensitive dye (VSD) cortical imaging over a large expanse of dorsal neocortex in mouse, we characterize sensory responses and their associated propagating waves.

To further characterize putative network adaptations after peripheral neuropathy, we leverage functional connectivity analysis in spontaneous activity as these interregional activity changes also represent recurrent interactions within cortex and moreover align with human fMRI findings. In human studies, alterations in the structure of spontaneous brain activity associated with allodynia are partially overlapping yet unique from the pain networks activated in response to evoked stimuli^22–24^. The observed alterations are centered on the default mode network (DMN), of which midline regions of cortex including the cingulate and medial prefrontal cortex in particular showing decreased connectivity to other nodes in the DMN. This reorganization is thought to accompany the development of chronic pain^25,26^. Emerging fMRI data in mouse suggests that midline cortical regions consistent with the murine DMN^27^ demonstrate altered functional connectivity after peripheral neuropathy^21^. Here, we build on this literature by leveraging the temporal resolution of VSD and a wide field of view to examine alterations in functional connectivity.

## Results

**Figure 1:**
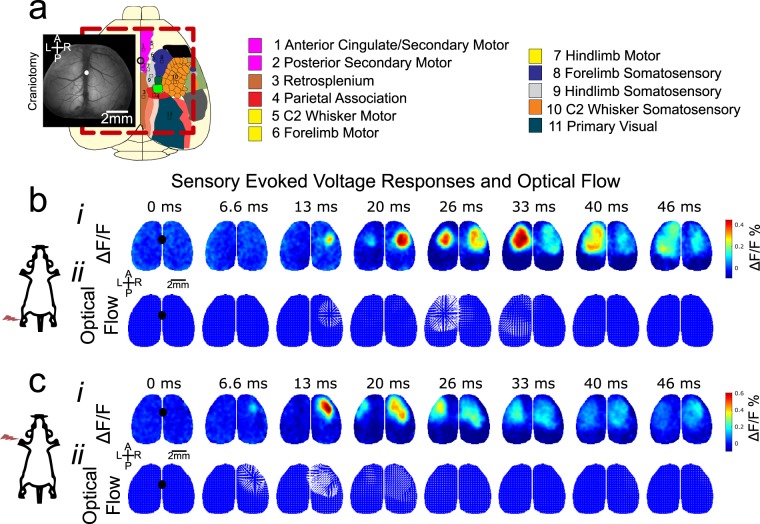
Voltage sensitive dye mesoscale cortical imaging. (**a**) A representative image of the exposed cortex after bilateral craniotomy and dura removal. At left the schematic represents cortical topography of the regions of interest. (bi) A representative time-course of the voltage responses, measured as a percentage change in fluorescence relative to baseline in a sham animal. Hindlimb stimulation results in a primary response in the contralateral hemisphere that spreads across the cortical surface, with a secondary response in the ipsilateral hemisphere. (bii) A representative time-course of optical flow, as measured by the frame-to-frame change accounted for by virtual pixel movement. Both the primary response and the secondary response elicit outward moving optical flow. Arrows represent relative magnitude and direction of optical flow. Each optical flow figure represents the forward-looking change between the above frame and the subsequent frame. (ci) Forelimb stimulation results in a primary response in the contralateral hemisphere that spreads across the cortical surface, with a secondary response in the ipsilateral hemisphere. (cii) As in hindlimb stimulation, propagation of the primary response results in transient optical flow outwards from the primary somatosensory region.

A large bilateral craniotomy exposed numerous cortical regions, including primary and secondary somatosensory and motor regions of the hindlimb, forelimb, and whisker, along with the anterior and midline association cortices: anterior cingulate (aM2/AC), secondary motor (pM2), retrosplenial (RS), and parietal association (PtA) cortices. (Fig. 1a). Hindlimb (Fig. 1bi) and Forelimb (Fig. 1ci) sensory stimulation via electrical stimulation produced a characteristic voltage response that originated in the primary somatosensory region in the hemisphere contralateral to the stimulated limb. Over tens of milliseconds, the voltage response was observed propagating outward from the primary region, along with a secondary voltage response in the primary somatosensory region of the ipsilateral hemisphere. Optical flow (represented by vector arrows in Fig. 1bii,cii) captures the propagation of voltage fluctuations as they spread over the cortex. It is important to note that localized voltage fluctuations centered around a maxima without generating a propagating wave are not associated with such vectors.

**Figure 2:**
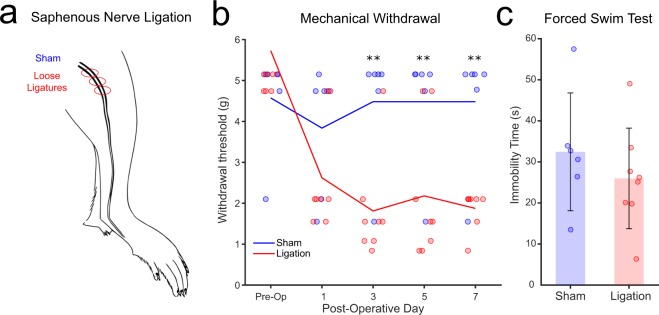
Saphenous nerve ligation results in mechanical hyperalgesia. (**a**) Schematic of the location of the saphenous nerve and ligations. No ligations were inserted in sham surgery. (**b**) Mechanical withdrawal as assessed with von Frey Filaments. Ligated mice (n = 8, red) showed a decrease in withdrawal threshold relative to sham (n = 6, blue) starting on day 3 post-operative. (**c**) Ligated mice showed no change in affective state relative to sham as assessed by immobility time in the forced swim test. **Indicates p < 0.01.

Saphenous nerve ligation (Fig. 2a) produced sustained hyperalgesia as assessed by mechanical withdrawal threshold to von Frey filaments applied to affected limb relative to sham. While withdrawal thresholds were similar at baseline, following surgery ligated animals (n = 8) showed a significant sensitization of withdrawal relative to (n = 6) sham (two-way RM ANOVA significant group effect, F(1,12) = 6.885, p = 0.022, and effect of day F(4,12) = 8.659, p < 0.001, significant interaction F(4,12) = 7.484, p < 0.001, follow-up pairwise comparisons p < 0.01 on days 3, 5, and 7; Fig. 2b). Rodent models of depression can produce broad alterations in cortical connectivity^28^, which may confound changes in cortical dynamics. To confirm that nerve ligation did not produce an emotional phenotype that might account for any observed changes, ligated and sham mice were assessed on the forced swim test, a standard assay of active avoidance that is altered in rodent models of depression^29^. No alteration was observed (t(12) = 0.910, p = 0.381; Fig. 2c).

**Figure 3:**
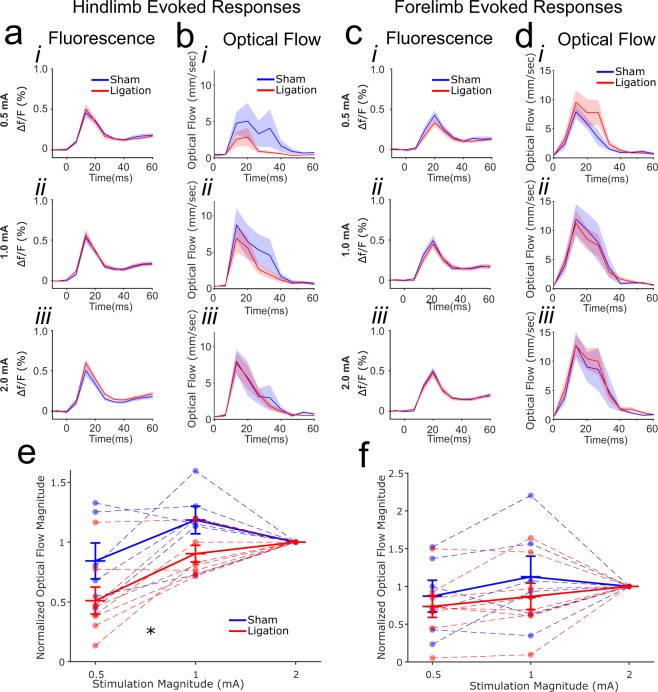
Hindlimb and forelimb sensory evoked responses and optical flow. (**a**) stimulation of the affected hindlimb produced no alteration in ligated mice (n = 8) relative to sham (n = 6) in fluorescence within the primary hindlimb somatosensory region in the hemisphere contralateral to the affected limb (5 × 5 pixel ROI) at 0.5 (i) 1.0 (ii) or 2.0 mA (iii), though the higher stimulation intensities evoked a significantly greater response (p < 0.001). (**b**) The aggregate optical flow in the same contralateral (primary) hemisphere was highly variable between animals, with no significant difference between groups, though greater optical flow was observed at higher stimulation intensities (p < 0.05). (**c**) Stimulation of the unaffected forelimb showed no alteration in fluorescence within the primary forelimb somatosensory region (5 × 5 pixel ROI). (**d**) Aggregate optical flow within the contralateral (primary) hemisphere was unchanged between groups. (**e**) Normalizing within subjects to the highest (2.0 mA) stimulation revealed significantly reduced optical flow in ligated mice relative to control in response to hindlimb stimulation. (**f**) No alteration in normalized optical flow was observed in response to forelimb stimulation in the contralateral (primary) hemisphere. *Indicates p < 0.05 main effect of group.

Under light isoflurane anesthesia (1%), electrical stimulation of the forepaw and hindpaw elicited characteristic voltage responses that originated in the primary somatosensory region and propagated outward, with a slightly delayed secondary response in the homotopic primary cortical somatosensory region. To assess whether nerve ligation resulted in alterations in the primary evoked response, fluorescence change (% relative to baseline) within a 25 pixel ROI centered on the primary hindlimb or forelimb sensory region was averaged for each frame and revealed a characteristic primary evoked response (Fig. 3a,c). At each stimulation intensity, peak responses from ligated and sham mice were compared within the first 50 ms following stimulus delivery in the contralateral (primary) hemisphere. With forelimb stimulation, no effect of treatment, stimulation intensity, or interaction was observed (two-way RM ANOVA, treatment effect F(1,12) = 0.531, p = 0.48, effect of stimulation magnitude F(2,12) = 1.268, p = 0.299, interaction F(2,12) = 0.426, p = 0.658). With hindlimb stimulation, no effect of treatment or interaction was observed (treatment effect F(2,12) = 0.292, p = 0.599, interaction F(2,12) = 1.565, p = 0.230). An effect of stimulation magnitude was observed (F(2,12) = 15.730, p < 0.001), with the 0.5 mA stimulation evoking significantly lower peak fluorescence relative to the higher stimulation intensities (ps < 0.001, bonferroni corrected pairwise comparisons).

To assess whether saphenous nerve ligation altered propagating waves of activity following sensory stimulation, we characterized the optical flow of VSD signals following sensory stimuli. The rise and fall of dF/F signal in the primary response only captures a portion of stimulus evoked dynamics. This primary response is accompanied by a time dependent spatial expansion of activity via horizontal fibers, the trajectory of which can be described as vector fields capturing velocity and direction from activity sources. We hypothesized that cortical remodeling can disrupt the flow of these time dependent signals, and therefore utilized optical flow methods to describe the temporal speeds of pixels within the traveling wave of activity accompanying the primary sensory-evoked response. These methods are extensively used in human electroencephalographic data^30^, primate cortical multi-electrode arrays^31^, and VSD signals from murine cortex^32,33^. Consistent with a travelling wave, optical vectors emerge after the primary sensory response to represent its outward propagation from the primary sensory cortical region. At each stimulation magnitude within the same hemisphere as the primary response (contralateral to the stimulated limb), aggregate optical flow from ligated and sham mice were compared over the first 50 ms following stimulation (Fig. 3b,d). No effect of treatment or interaction with stimulation intensity was observed with forelimb stimulation (two-way RM ANOVA, treatment effect F(2,12) = 0.115, p = 0.740, interaction F(2,12) = 0.358, p = 0.703). A marginal effect of stimulation magnitude was observed, with higher stimulation intensities evoking more optical flow (F(2,12) = 3.061, p = 0.065). With stimulation of the affected hindlimb, no effect of treatment or interaction was observed (treatment effect F(2,12) = 0.549, p = 0.473, interaction F(2,12) = 2.481, p = 0.105). A significant effect of stimulation magnitude was observed (F(2,12) = 8.322, p = 0.002), with the 0.5 mA stimulation evoking significantly less optical flow relative to higher stimulations (ps < 0.05, bonferroni corrected pairwise comparisons).

The average optical flow magnitude across the hemisphere peaked at approximately 10 mm/sec, however calculating peak optical flow speed for each pixel revealed a wide range of peak magnitudes (Supplementary Fig. 1). At 1 mA or 2 mA stimulation magnitudes, the highest optical flow magnitudes averaged ~40 mm/sec in the contralateral (primary) hemisphere, with individual peak magnitudes greater than 90 mm/sec in certain animals. These speeds are consistent with previous estimates of mesoscopic travelling waves^12,33^. Comparing between groups revealed no significant difference in the distribution of peak optical flow magnitudes.

We observed highly variable absolute values of optical flow vectors between subjects. To correct for this variability and ask whether nerve ligation may produce a subthreshold alteration in sensory evoked optical flow, we normalized aggregate optical flow over the same 50 ms time window to the highest (2 mA) stimulation magnitude for each animal (Fig. 3e,f). Saphenous nerve ligation resulted in significantly reduced normalized optical flow in response to hindlimb stimulation on the contralateral (primary) side (effect of magnitude F(1,12) = 17.352, p < 0.001; group effect F(1,12) = 5.438, p = 0.038; ns interaction p = 0.778, Fig. 3e) and on the ipsilateral side (Supplementary Fig. 2). No alteration was found for stimulation of the cortical representation of the unaffected forelimb in the contralateral (primary) hemisphere (effect of magnitude F(1,12) = 4.539, p = 0.055; group effect F(1,12) = 0.556, p = 0.470; ns interaction p = 0.507, Fig. 3f) or the ipsilateral hemisphere (Supplementary Fig. 2). Thus when controlling for inter-subject variability, optical flow was significantly reduced in ligated animals, suggesting a threshold change in the propagation of activity across the cortex. Normalizing peak fluorescence within animal produced no group effect in either forelimb or hindlimb stimulation (Supplementary Fig. 2).

As hemisphere-wide alterations could be driven by regionally specific changes in optical flow, we partitioned the hemisphere contralateral to the injury into a primary response region and a surround region. The primary response region was defined by the set of contiguous pixels that shared a peak response latency with the maximal df/F response in the 5 × 5 pixel primary ROI. The size of this region differed between animals and stimulation magnitudes but not between groups (data not shown). Remaining pixels were assigned to the surround region (Supplementary Fig. 3c,d). Examining normalized optical flow as above within these regions revealed that ligation specifically reduced optical flow in the surround region, while optical flow within the primary response region was unaffected (Supplementary Fig. 3a,b). Thus the hemisphere-wide reduction in optical flow we observed was attributable to a reduction in optical flow magnitude in distal regions rather than regions within or close to the primary hindlimb somatosensory region.

An alternative method for assessing propagating waves of activity is to examine the temporal offset of peak responses^34^, which creates a phase latency map of peak response times (Supplementary Fig. 4a,b). Approximately concentric regions of increasing phase latency illustrated the presence of travelling waves. To examine the effect of nerve ligation on evoked activity, both the area and average peak df/F value were compared for hindlimb and forelimb at the three stimulation intensities (Supplementary Fig. 4c–f). While no alteration in average df/F was observed in any condition, a specific reduction in the area with a phase offset response was observed with 0.5 mA hindlimb stimulation. This is consistent with our observations using optical flow magnitude, wherein regions outside a primary response region show reduced propagation of activity triggered by stimulation of the affected limb.

**Figure 4:**
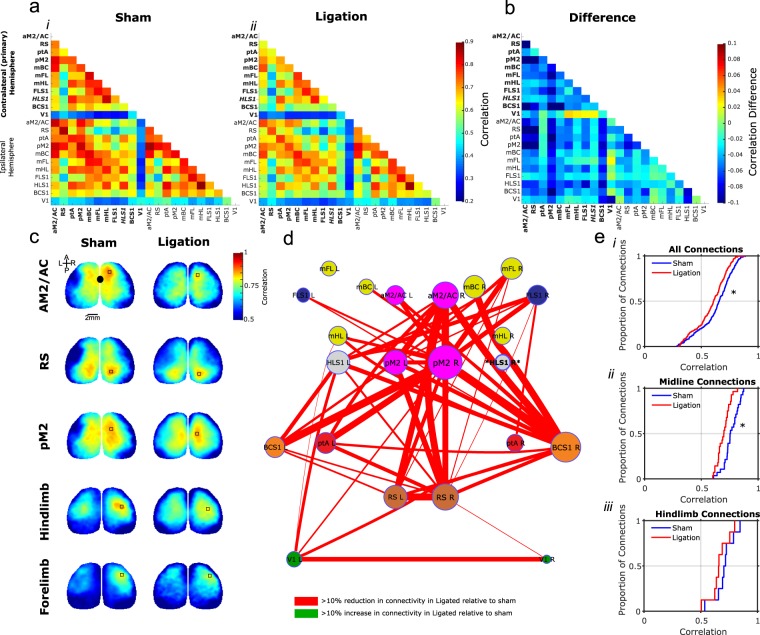
Functional connectivity during spontaneous activity is significantly reduced. (**a**) Average zero-lag correlation matrices between 22 ROIs (11 per hemisphere) in sham (i, n = 6) and ligated (ii, n = 8) mice. (**b**) A difference matrix between sham and ligated mice shows widespread reduction in zero-lag correlation. (**c**) Similarly, seed pixel correlation maps in five notable regions (AM2/AC, secondary anterior motor cortex, anterior cingulate;RS, Retrosplenial cortex;pM2, secondary posterior motor cortex; Hindlimb, primary hindlimb somatosensory region; Forelimb, primary forelimb somatosensory region) show reduced correlation. Bolded region in a,b,c indicates affected region. (**d**) An undirected graph of connectivity changes shows widespread reductions in connectivity. Bolded region indicates affected region. (**e**) An overall reduction in connectivity is evidenced by a significant effect of group in a general linear mixed effects model on all connections (i), as well as specifically the midline connections between aM2/AC, pM2, RS and ptA, parietal association area (ii). The connections with the affected hindlimb somatosensory region were unaffected (iii). *Indicates p < 0.05.

As optical flow reflects recurrent connections within cortex, we sought to corroborate these findings using interregional functional connectivity based on zero-lag Pearson correlation calculated from spontaneous activity in a 30-minute recording session, which also reflects large scale functional networks and recurrent connections within the brain. Spontaneous cortical activity shows large scale structured activity dynamics observable with VSD imaging during light anesthesia^32,35^. Consistent with optical flow analyses that suggested the effects of nerve ligation were widespread, functional connectivity analysis revealed a broad reduction in connectivity between various regions. These are qualitatively illustrated in Fig. 4c with seed-pixel correlations, and the interregional correlation structure underlying this effect is illustrated in Fig. 4a,b. To better visualize these data, we utilized graphical representation with nodes organized according to their anatomical location (Fig. 4d). Using a GLMEM, we confirmed that there was a global decrease in functional connectivity in ligated animals as compared with sham, (231 connections/animal, t(3232) = 2.362, p = 018, Fig. 4e,i). Considering only midline regions (aM2/AC, pM2, RS, PtA) revealed highly reduced connectivity (28 connections/animal, t(390) = 2.49, p = 0.013, Fig. 4eii). Interregional connectivity with the affected hindlimb region was not specifically reduced (21 connections/animal, t(292) = 1.13, p = 0.258, Fig. 4eiii). Power was non-significantly reduced in the slow band, although a marginal effect was observed (Supplementary Fig. 5). To confirm this alteration was not a specific product of anesthesia, functional connectivity was also assessed during a 5-minute awake, head fixed imaging session (Supplementary Fig. 6). While midline regions were similarly reduced, overall functional connectivity was not significantly reduced as was observed with the longer 30-minute recording session under 1% anesthesia.

## Discussion

We report a novel alteration in sensory processing in cortex after experimental peripheral neuropathy. Despite intact primary response magnitude in the hindlimb primary somatosensory region of the hindlimb in ligated animals, we find evidence for reduced propagating waves using optical flow methods in response to hindlimb stimuli, particularly at lower stimulus intensities. Attempts to corroborate alterations to recurrent cortical connections using spontaneous activity functional connectivity, in turn, revealed large scale reductions in functional connectivity that were not restricted to the hindlimb primary somatosensory region.

To our knowledge, this is the first characterization of propagating waves of activity associated with sensory responses in peripheral neuropathy. We observed altered sensory-evoked dynamics after evoked stimulation that was intensity dependent, such that weak hindlimb stimulation elicited a markedly diminished propagating wave. As voltage-sensitive dye allows direct measurement of bulk voltage fluctuations in cortical tissue, which is dominated by synaptic events in dendrites occupying superficial cortical layers, VSD imaging is well suited to capturing this component of cortical sensory-evoked activity using optical flow analysis^33^. While the function of these waves is unclear, our data suggests that adaptation after the injury serves to constrain horizontal propagation of activity by horizontal fibres in superficial cortical layers^12^. Intracortical propagation of activity along horizontal fibres is heavily shaped by feedforward inhibition and excitatory/inhibitory (E/I) balance in feedforward circuits^36^, and nerve ligation has been recently shown to enhance feedforward inhibition in mouse PFC^37^.

Hyperexcitability in the affected regions of somatosensory cortex has been reported in experimental models of neuropathy^19,38,39^ something that we did not observe using VSD imaging and the saphenous nerve injury model. It is unclear whether this discrepancy is best attributed to differences in the experimental model of neuropathy^19,38,39^ or to anesthetic agent^38,39^. The human literature in chronic pain is similarly mixed. Many studies report reduced electroencephalography somatosensory event related potentials compared to control participants^40^, while other studies utilizing fMRI reveal enhanced cortical responses^41–43^. These findings highlight the complexity and heterogeneity of peripheral neuropathies and potential dissociation of the primary sensory evoked response from a hyperalgesic/allodynic phenotype. Accordingly, self reports of pain intensity correlate more strongly with activation within affective brain rather than primary sensory regions^23^.

Using inter-regional correlation of activity, we identified broad changes in functional connectivity consistent with large scale reorganization after peripheral neuropathy. In human fMRI, similar alterations in somatosensory regions have been reported^19,38,39^, however large changes are simultaneously observed in midline regions implicated in emotional regulation^44–46^. Indeed, we observed a widespread reduction in functional connectivity during spontaneous activity, with the largest reductions localized to midline regions that are homologous to default mode network (DMN) hubs in the human brain^27,47^. This aligns with studies in humans^22,24^, yet the converse has also been reported^26,48–53^ with DMN hyperconnectivity reported. An important confound in human investigations is that chronic pain is highly comorbid with affective disorders^54^, which are themselves associated with hyperconnectivity in the DMN^55^. Our data dissociates these potentially confounding influences, as animals were imaged prior to developing a depressive-like phenotype.

Our data and analyses are subject to limitations and caveats. We acquired both sensory evoked activity and spontaneous activity under light isoflurane anaesthesia, and while this has certain advantages by mitigating sensorimotor confounds or planning, its relevance to the awake behaving animal remains to be confirmed. Isoflurane anesthesia attenuates sensory responses^32^, however we attempted to mitigate this potential confound by normalizing response and propagating wave magnitude within subjects to a ceiling stimulation intensity (2 mA). With respect to resting state connectivity, conserved structure of spontaneous activity is observable in quiet wakefulness, sleep, and anaesthesia^56,57^, including using mesoscale cortical VSD imaging^32,35,58^. Finally, this acute preparation prevented us from repeated sampling to quantify change over time, however novel genetically encoded voltage indicators may permit such approaches in the future^59^.

Succinctly, we find several indices of cortical reorganization after peripheral neuropathy that transcend primary sensory regions and influence sensory-evoked activity. Identifying the central nodes of this reorganization is a crucial next step which could lead to novel therapeutic strategies based on non-invasive neurostimulation^60^. In addition, our observation of altered spontaneous connectivity within the slow power band suggest a novel biomarker for neuropathic pain that could aid in diagnosis and guide treatment strategies^61^.

## Methods

### Animals

Adult male C57BL6J mice (N = 14, 3–4 months old) were used for all experiments. Animals were group housed on a 12:12 light cycle with *ad libitum* access to food and water. All procedures were approved by the University of British Columbia Animal Care Committee (UBC ACC) in accordance with the ethical standards set forth by the Canadian Council on Animal Care (CCAC).

### Saphenous nerve ligation

To model focal neuropathic pain, the saphenous nerve of the right hindlimb was ligated ten days prior to imaging^14^. Mice were anesthetized with isoflurane (5% induction, 2% maintenance in 1 L/min O_2_) and administered buprenorphine (SC, 0.05 ml, 0.3 mg/ml) for analgesia. Body temperature was maintained at 37 °C with a thermometer feedback controlled heatpad. The anterior aspect of the left hindlimb was shaved and disinfected with betadine and alcohol (3x alternating), and an 8 mm incision was made overlying the saphenous triad. Subcutaneous and connective tissue was blunt dissected to isolate the saphenous nerve and 8-0 braided suture (Vicryl, Ethicon Inc., USA) was tied loosely around the nerve at 1 mm intervals. Sham animals underwent the same incision and dissection surgical procedure but no ligation was performed. Subsequent to ligation, the primary incision was closed (5-0 Vicryl, Ethicon Inc., USA) and mice were recovered and returned to their homecage.

### Mechanical withdrawal assay

Mechanical withdrawal threshold was assayed at several points using von Frey Filaments^62^. Mice were restrained within a modified restraint tube with a metallic mesh base that allowed Von Frey filament application to the hindpaw. To determine the mechanical threshold, von Frey filaments ranging from 0.5 g to 6 g were applied to the plantar surface of the hindpaw. Beginning with the weakest filament, filaments were applied three to five times for two seconds. The animal’s response to filament application was observed, with a positive response defined as withdrawing, lifting, holding, or shaking the paw. The mechanical pain threshold was defined as ≥3 out of 5 positive applications. For subsequent assessments, withdrawal threshold was assessed using an up-down assay at the withdrawal threshold determined in the previous assessment for each animal.

### Forced swim test (FST)

Mice were placed in a transparent glass beaker (25 cm height, 18 cm diameter), containing water at 24-5 °C. For 5 minutes, the mice remained in the water while an observer coded their active swimming and floating (including efforts to maintain position). The water was changed between animals. Only the final 4 minutes of the experiment are reported.

### VSD Imaging surgery

Ten days after nerve ligation, mice were anesthetized with isoflurane (5% induction, 1.5% maintenance) and prepared for bilateral cortical VSD imaging as previously detailed^58,63^. Recording of experimental groups was interleaved. Briefly, a screw was fastened to the exposed skull with cyanoacrylate and dental cement for subsequent head fixation, and an approximately 7 × 8 mm bilateral craniotomy was performed to expose the cortical surface 3.5 mm anterior to bregma to 4.5 mm posterior to bregma and 4 mm lateral to bregma. The underlying dura was removed to facilitate dye penetration and enhance optical clarity. RH1692 (Optical Imaging, New York, NY) was dissolved in HEPES buffered saline (1 mg/ml) and applied to the cortex for 60–90 minutes. Following washing of unbound dye with HEPES buffered saline, the brain was covered with 1.5% agarose and sealed with a glass coverslip to minimize respiration artifacts. This preparation reveals a wide expanse of neocortex including sensory, motor, and midline association regions (Fig. 1a). Dye incubation results in penetration throughout all neocortical layers^64^.

### VSD imaging protocol

Mice were head fixed for imaging under light anesthesia (1% isoflurane) with body temperature maintained at 37 °C with a feedback thermistor. 12-bit images were captured with 67um/pixel resolution at 50 Hz for spontaneous activity or 150 Hz for sensory evoked activity with a CCD camera (1M60 Pantera, Dalsa, Waterloo, ON) and EPIX E4DB frame grabber using XCAP 3.8 imaging software (EPIX, Inc., Buffalo Grove, IL) through a macroscope composed of front-to-front video lenses (8.6 × 8.6 mm FOV, 67 µm/pixel). VSD was excited with a red LED (Luxeon K2, 627 nm) filtered at 630 nm +−15 nm and fluorescence captured using a 673–703 nm bandpass optical filter (Semrock, New York, NY). Imaging was focused into the cortex to a depth of ~1 mm to reduce VSD signal distortion from large blood vessels. Ambient light resulting from VSD excitation was measured at 8.65e-3 W/m2. With dye incubation throughout all cortical layers, and diffraction of excitation and emission signals, the bulk of the fluorescent signal originates from neuropil in superficial layers of the cortex^64^. Spontaneous activity was sampled at 50 Hz for 30 minutes (90,000 frames). For anesthetic free imaging, mice were recovered with oxygen (1 L/min) while head-fixed for 15 minutes, and awake head-fixed spontaneous activity was recorded at 150 Hz for 5.5 minutes (50,000 frames).

### Sensory evoked responses

Sensory evoked responses were imaged at 150 Hz for 108 frames (720 ms) in response to forelimb and hindlimb stimulation under light anesthesia (1% isoflurane). For stimulation, 0.14 mm acupuncture needles were inserted into the left paws and 0.5, 1 and 2 mA square pulses (1 ms) were delivered. For both modalities, 10 stimulations in addition to 5 no-stimulation trials were delivered, with an interstimulus interval of 10 s.

### Data analysis

VSD image stacks were analyzed using custom-written MATLAB code (Mathworks, MA). For sensory evoked activity, 10 trials were averaged to minimize the effects of spontaneous fluctuations and normalized to a 5 trial average of blank trials. Photobleaching artifacts were removed by using a detrending filter based on the cross pixel time-varying average signal and the per-pixel average fluorescence across the trial. All individual pixel signals were then expressed as a relative change (ΔF/F × 100%) and normalized to the pre-stimulus baseline. A 5 × 5 pixel ROI was selected based on the centroid of peak response for each sensory modality, and secondary sensory modality and association area ROIs were interpolated based on these sensory ROIs and standard stereotaxic coordinates. For spontaneous activity analysis, per-pixel fluorescence fluctuations were first filtered in the slow activity band (0.5–6 Hz) and then expressed as relative change (ΔF/F × 100%).

### Optical flow analysis

Optical flow was calculated using the combined local-global (CLG) algorithm of optical flow estimation from the Optical Flow Analysis Toolbox^33^, producing a per-pixel estimation of the optical flow between image frames. In order to capture broad alterations in optical flow irrespective of direction, the magnitude of optical flow vectors from every pixel in each hemisphere was averaged at each time point, producing a time-varying signal that peaked shortly after sensory stimulation, which we refer to as aggregate optical flow. Optical flow magnitude was integrated over the 10 frames following stimulation (66 ms) to produce a single estimate of optical flow magnitude (integrating across the entire trial did not alter results).

The peak optical flow magnitude was found per pixel for each hemisphere to examine alterations in peak optical flow. A cumulative distribution of peak pixel magnitudes was created for each animal and averaged across animals for each stimulation magnitude.

To test whether hemisphere-wide alterations in normalized optical flow were driven by regionally specific alterations, per-pixel responses were segregated into a primary response region and a surround region. Regions were defined based on peak phase latency relative to stimulus onset, as in the analysis detailed below. The primary region was defined as contiguous pixels whose peak df/F value corresponded to the peak within the 5 × 5 pixel ROI of the primary response, while the remaining pixels were assigned to the surround region. Optical flow magnitudes within these defined regions were averaged for each frame and integrated across 10 frames as above.

### Peak phase latency analysis

The temporal offset of peak activity was used as an alternative analysis of propagating activity^34^. Processed evoked ΔF/F was additionally filtered between 5 and 30 Hz on a per pixel basis, and the first local peak of activity after stimulation was found for each pixel. Contiguous areas with simultaneous peak latency were identified by a one step erosion/dilation for each latency followed by removal of minimally sized regions with peak responses at each frame. This resulted in typically one or two large contiguous regions, however any remaining regions were included in analysis, which was confined to the contralateral (primary) hemisphere. Total area was calculated by a sum of participating pixels, while average response magnitude was calculated from the mean df/F across pixels for each frame.

### Functional connectivity analysis

Correlation matrices were created based on the zero-lag Pearson correlation of ROI pairs filtered in the slow activity band (0.5–6 Hz) during 30 minutes of spontaneous activity acquired at 50 Hz filtered or 5.5 minutes of spontaneous activity in a period of quiet wakefulness. Individual ROIs were calculated for all animals based on interpolation from the peak responses to hindlimb, forelimb, and whisker sensory responses. To visualize network changes, average differences were plotted as an undirected network diagram using modified code from the Bioinformatics and Brain Connectivity Toolbox^65^. To facilitate visualization, each connection was only represented if the correlation value was 10% greater or inferior to that observed in the sham group. To test the statistical significance of functional connectivity differences between groups, we utilized generalized linear mixed effect models (GLMEM; Wilkinson notation: Inter-Regional_Correlation ~1 + Group + (1|Region) + (1|Mouse)) implemented in MATLAB.

## Supplementary information

Supplementary Figures

## Data Availability

The datasets generated and analysed for the current study are available from the corresponding author on reasonable request.
