# Supplementary material for: Peripheral Nerve Ligation Elicits Widespread Alterations in Cortical Sensory Evoked and Spontaneous Activity
Source: Sci Rep. 2019 Oct 25;9:15341. doi: 10.1038/s41598-019-51811-8 (PMC6814845; doi:10.1038/s41598-019-51811-8)

Supplementary Figures

Peripheral Nerve Ligation Elicits Widespread Alterations in Cortical Sensory Evoked and Spontaneous Activity.

Donovan M Ashby, Jeffrey LeDue, Timothy H Murphy, Alexander McGirr.

Supplementary Figure 1. Peak optical flow magnitude. Average cumulative distribution of peak optical flow magnitude for the contralateral (primary) hemisphere (a), and the ipsilateral hemisphere (b) in response to 0.5, 1 and 2 mA stimulation of the hindlimb. Example heatplots of the distribution of these peak magnitudes illustrates that the highest speed pixels surround the primary hindlimb somatosensory regions in each hemisphere (c). No difference between sham and control was observed in comparing these distributions with a GLMEM; t(60702)=1.024, p=.306; t(60702)=0.693, p=.488; t(60702)=0.284, p=.777 for 0.5, 1, 2 mA stimulation in primary hemisphere. t(60702)=1.377, p=.168; t(60702)=0.391, p=.695; t(60702)=0.793, p=.427 for 0.5, 1, 2 mA stimulation in ipsilateral hemisphere.

Supplementary Figure 2. Normalized peak fluorescence in contralateral (primary) hemisphere and optical flow in ipsilateral hemisphere. Peak fluorescence normalized to the highest (2 mA) stimulation magnitude within each animal is plotted for hindlimb (a) and forelimb (b) stimulation in the ROI for each sensory modality. No effect of ligation was observed when comparing normalized fluorescence with hindlimb (effect of magnitude F(1,12)=11.52, p=.005; group effect F(1,12)=1.616, p=.228; ns interaction p=.683) or forelimb stimulation (effect of magnitude F(1,12)=6.02, p=.03; group effect F(1,12)=0.93, p=.352; ns interaction p=.904). Below is normalized optical flow magnitude in the ipsilateral hemisphere in response to hindlimb (c) and forelimb (d) stimulation. Consistent with observations from the contralateral (primary) hemisphere, normalized optical flow is marked reduced in ligated animals relative to sham in response to hindlimb (effect of magnitude F(1,12)=14.396, p=.003; group effect F(1,12)=12.310, p=.004; ns interaction p=.851) but not forelimb stimulation(effect of magnitude F(1,12)=5.345, p=.039; group effect F(1,12)=0.644, p=.438; ns interaction p=.310).

Supplementary Figure 3. Regionally segregated optical flow magnitude. To test whether hemisphere-wide alterations in normalized optical flow were driven by regionally specific alterations, per-pixel responses were segregated into a primary response region and a surround region based on peak phase latency relative to stimulus onset, as in figure S4. The primary region was defined as contiguous pixels whose peak df/F value corresponded to the peak within the 5x5 pixel ROI (d), while the remaining pixels in the hemisphere were considered the surround region (c). Within-subjects normalization to the highest stimulation (2mA) of the aggregate average optical flow magnitude showed a specific reduction in the surround region (relative to sham), while no difference between groups was observed in the primary region. Repeated Measures ANOVA, Surround Region: F(1,12)=13.395, p=.003 effect of magnitude;F(1,12)=5.778, p=.033 effect of group;F(1,12)=.274, p=.610 no interaction. Repeated Measures ANOVA, Primary Region: F(1,12)=12.494, p=.004 effect of magnitude;F(1,12)=.009, p=.922 no effect of group;F(1,12)=.0169, p=.899 no interaction. * indicates p<.05.

Supplementary Figure 4. Latency to phase peak for hindlimb and forelimb stimulation. Cortical maps were generated from average evoked responses, and the latency from stimulation to the first local peak in df/F for each pixel was calculated. A contiguous region encompassing the primary hindlimb or forelimb sensory ROI had the shortest latency response, with increasing

delays observed at a distance from the primary response, indicative of a travelling wave (a,b). To compare the effects of nerve ligation, areas participating in the travelling wave were identified by a one step erosion/dilation followed by removal of minimally sized regions with peak responses at each frame. This resulted in typically one or two large contiguous regions, however any remaining regions were included in analysis, which was confined to the contralateral (primary) hemisphere. The area participating in the travelling wave was typically largest at 13 ms post-stimulation, however in ligated mice this area was smaller and peaked earlier with the lowest magnitude hindlimb stimulation (c,d).Two-way ANOVA, all group and group*time interaction ps>.05, except for hindlimb 0.5 mA stimulation, ($F(5,12)=25.78, p<.001$, effect of time; $F(1,12)=0.63, p=.442$, effect of group; $F(5,12)=2.402, p=.047$, group*time interaction; follow-up pairwise comparisons (bonferroni corrected) p=.016 effect of group at 20 ms post-stimulation). The average fluorescence peaked one frame earlier, at 6 ms, within the primary response region in all conditions, with no significant differences between groups (e,f). Two-way ANOVA, all group and group*stimulation interactions p>.05. * indicates p<.05.

Supplementary Figure 5. Slow bandpower (0.5-6 Hz) during spontaneous activity. Eleven cortical ROIs are plotted comparing power in the slow band between sham and ligated mice. Regions contralateral to the affected hindlimb, including the primary somatosensory region representing the ligated limb are represented as filled circles, while regions ipsilateral to the affected limb are represented by empty circles. Ligation marginally reduced power as indicated by a GLMEM, 22 regions/animal, $t(306)=1.76$, $p=.079$). AM2/AC, secondary anterior motor cortex, anterior cingulate; RS, retrosplenial cortex; ptA parietal area; pM2, secondary posterior motor cortex; mBC, whisker motor region; mFL, forelimb motor region; mHL, hindlimb motor region; FLS1, primary forelimb somatosensory region; HLS1, primary hindlimb somatosensory region; BCS1, primary whisker somatosensory region; V1, primary visual area.

Supplementary Figure 6. Functional connectivity in the awake, head fixed state. Spontaneous activity was recorded at 150 Hz for 5.5 minutes. Fluorescence signals (df/F) were filtered between 0.5-6 Hz, and a zero-lag correlation matrix was calculated for 22 ROIs in sham (n=6) and ligated (n=7) mice (a). As in the isoflurane anesthetic state, functional connectivity was broadly reduced as illustrated by an undirected graph of connectivity changes (b). While an overall reduction in connectivity was not observed in a general linear mixed effects model on all connections (i, $t(3001)=1.39$, $p=.165$), the midline connections between aM2/AC, pM2, RS and ptA, parietal association area were significantly reduced as in the anesthetized state (ii, $t(362)=2.03$, $p=.043$). The connections to the affected hindlimb somatosensory region were unaffected (iii, $t(271)=0.725$, $p=.469$). * indicates p<.05.

Supplementary Figure 1. Ashby et al., Peripheral Nerve Ligation Elicits Widespread Alterations in Cortical Sensory Evoked and Spontaneous Activity.

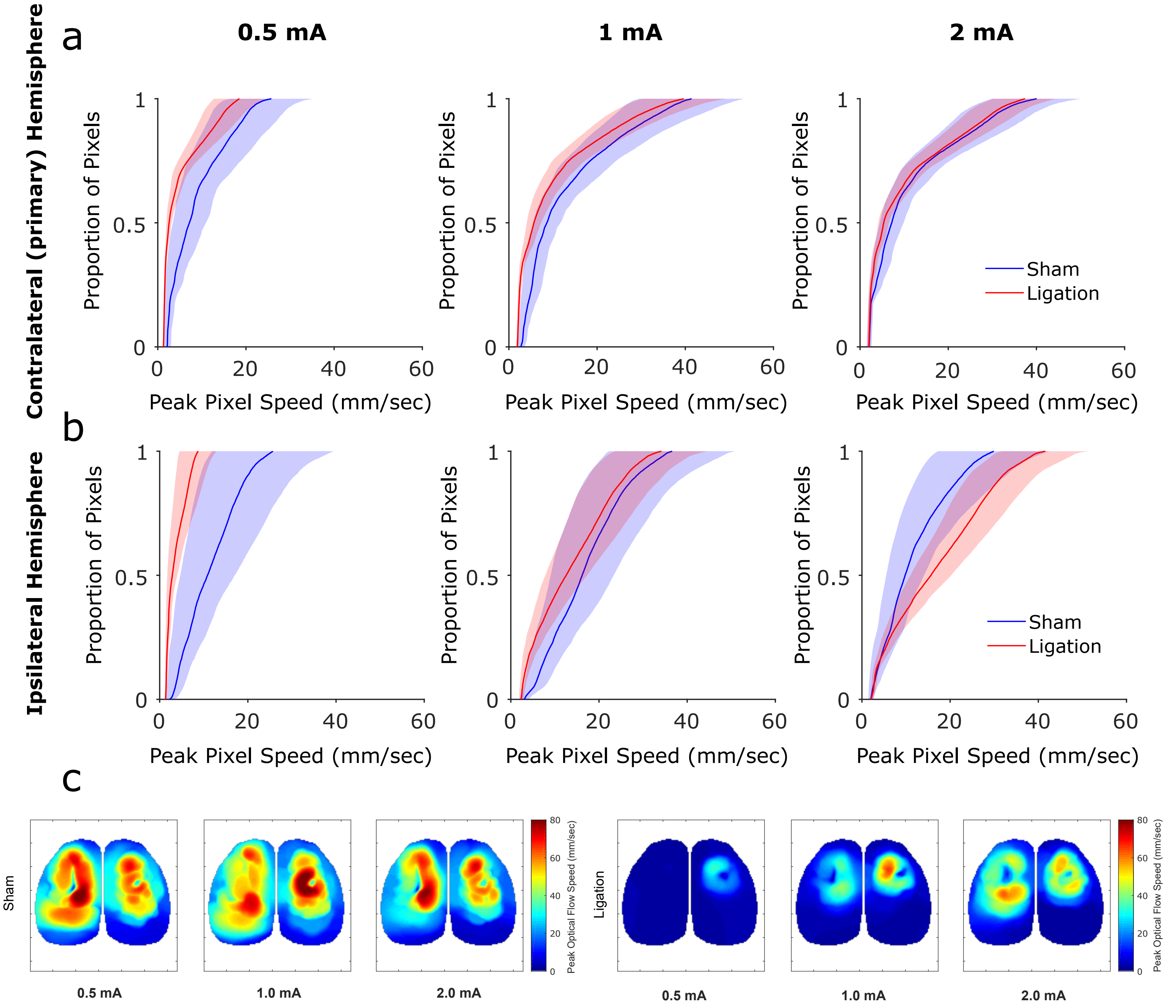

Supplementary Figure 2. Ashby et al., Peripheral Nerve Ligation Elicits Widespread Alterations in Cortical Sensory Evoked and Spontaneous Activity.

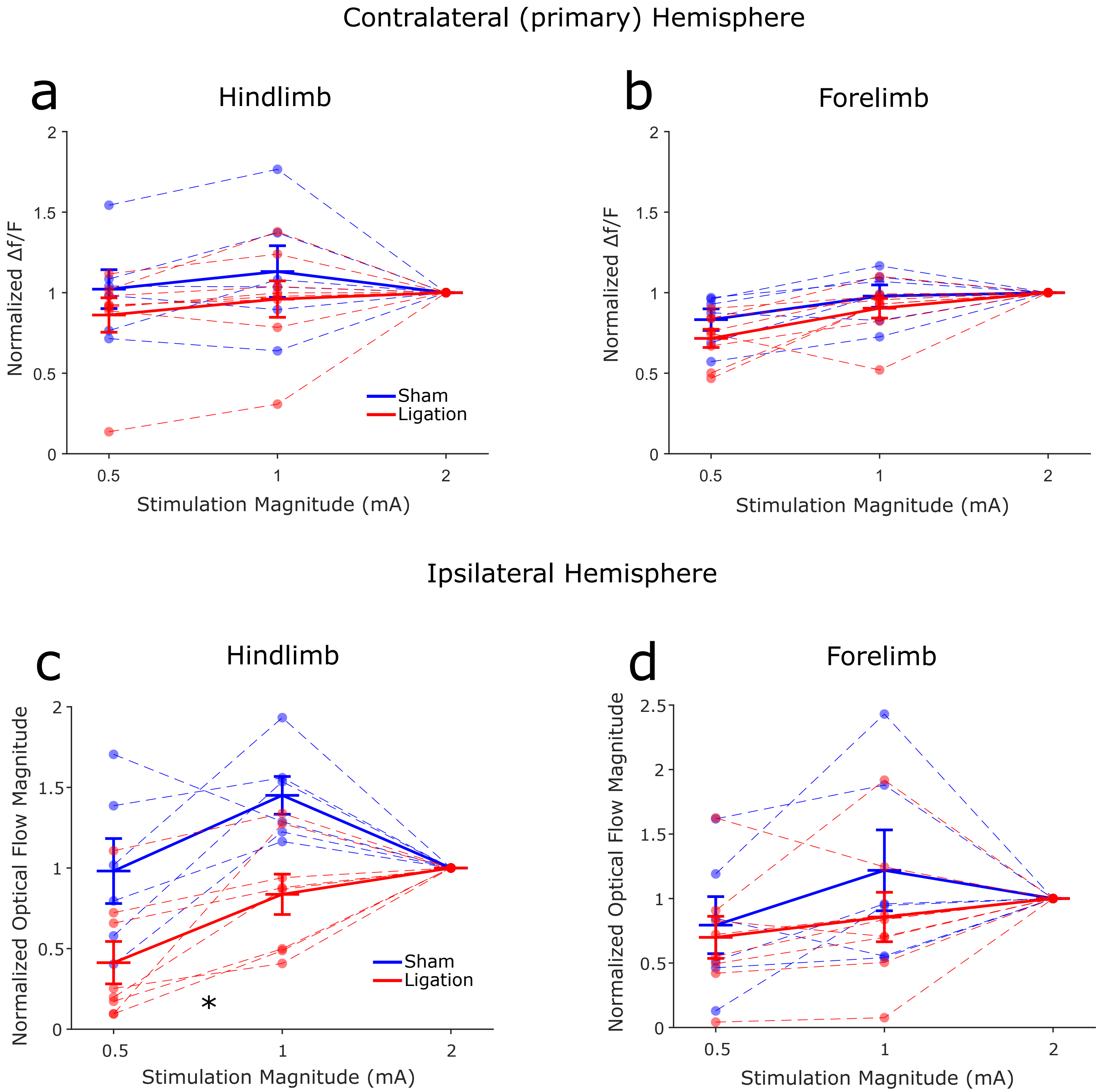

Supplementary Figure 3. Ashby et al.,
Peripheral Nerve Ligation Elicits Widespread Alterations in Cortical Sensory Evoked and Spontaneous Activity.

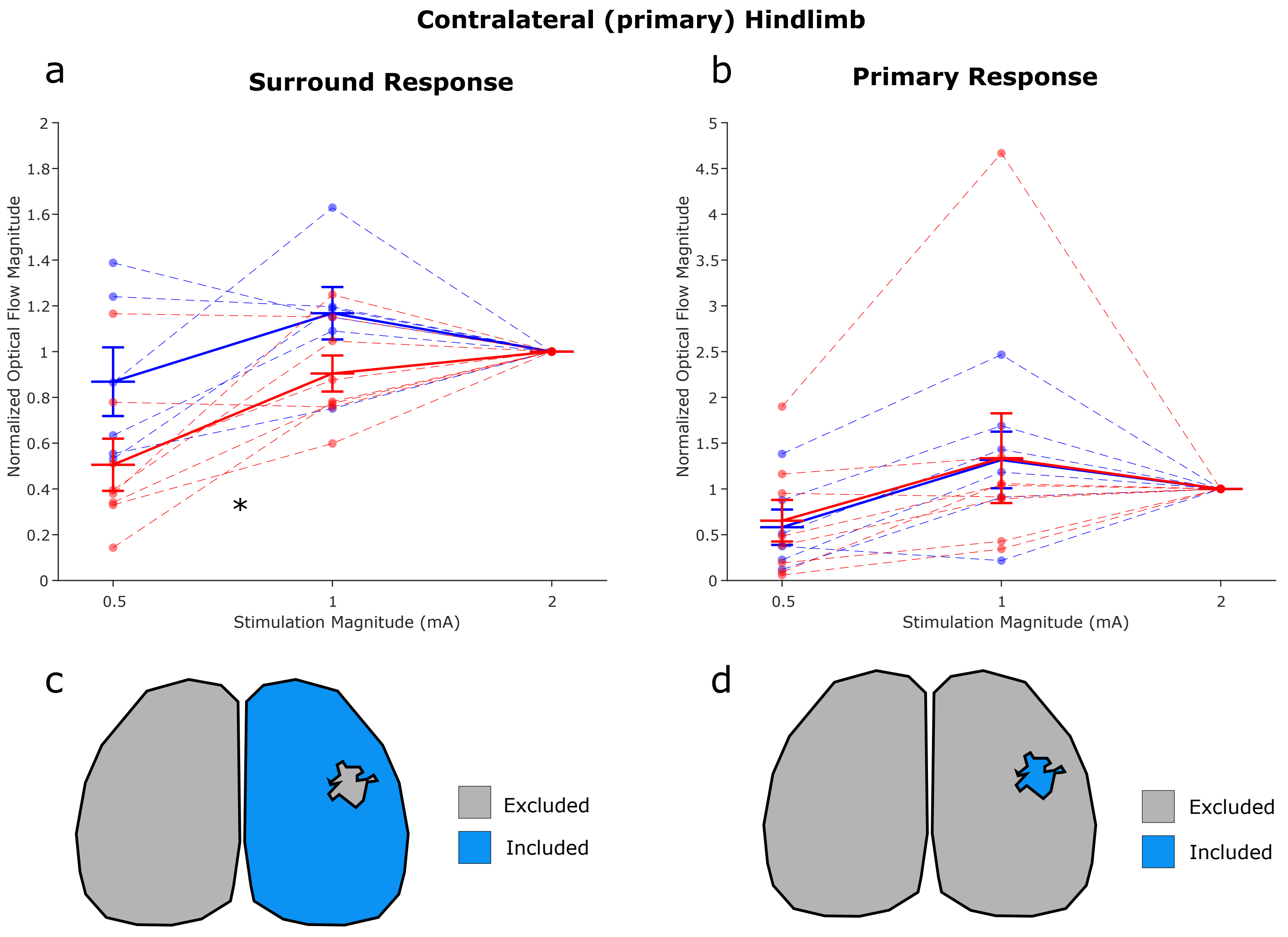

Supplementary Figure 4. Ashby et al., Peripheral Nerve Ligation Elicits Widespread Alterations in Cortical Sensory Evoked and Spontaneous Activity.

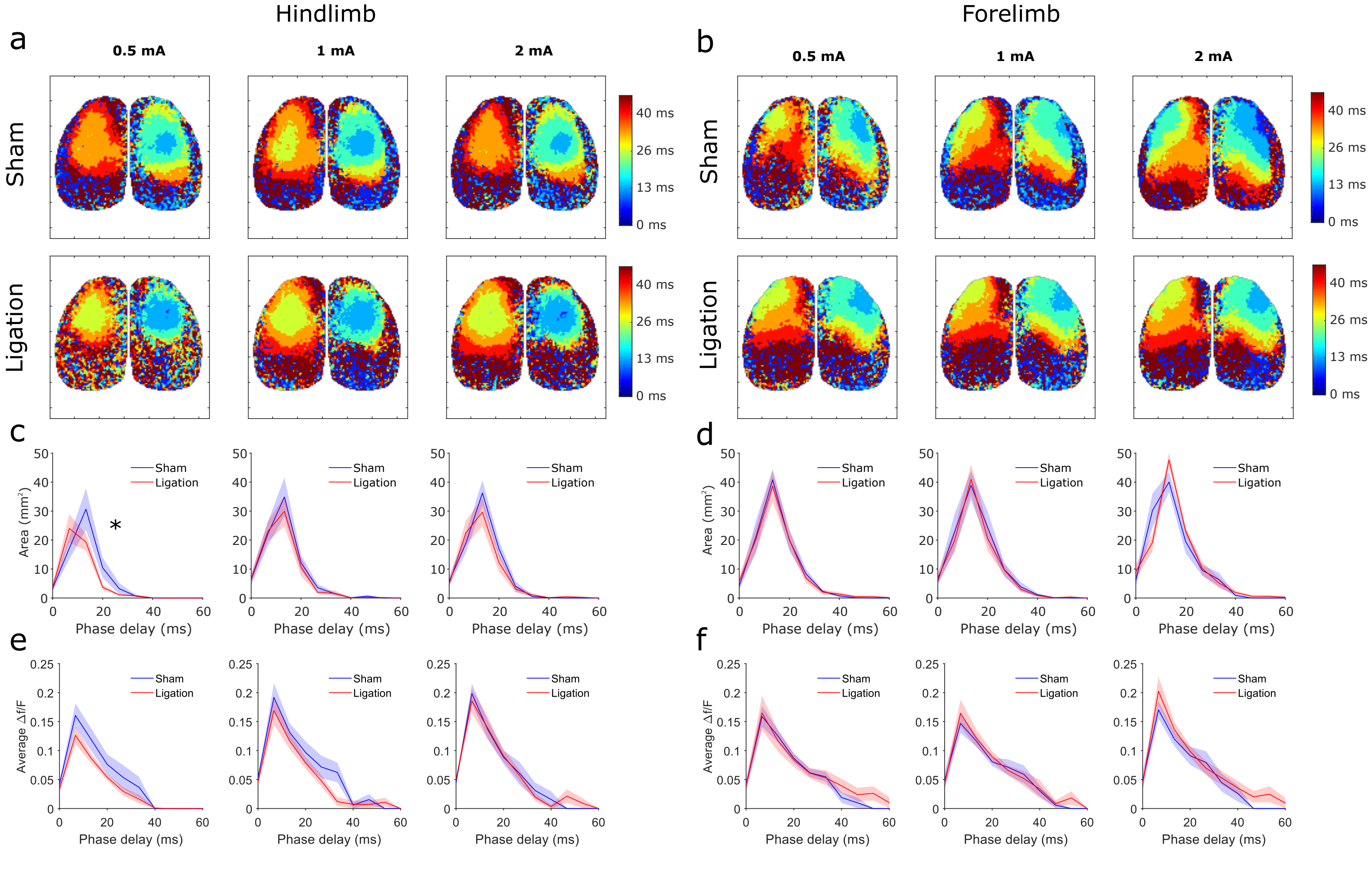

Supplementary Figure 5. Ashby et al., Peripheral Nerve Ligation Elicits Widespread Alterations in Cortical Sensory Evoked and Spontaneous Activity.

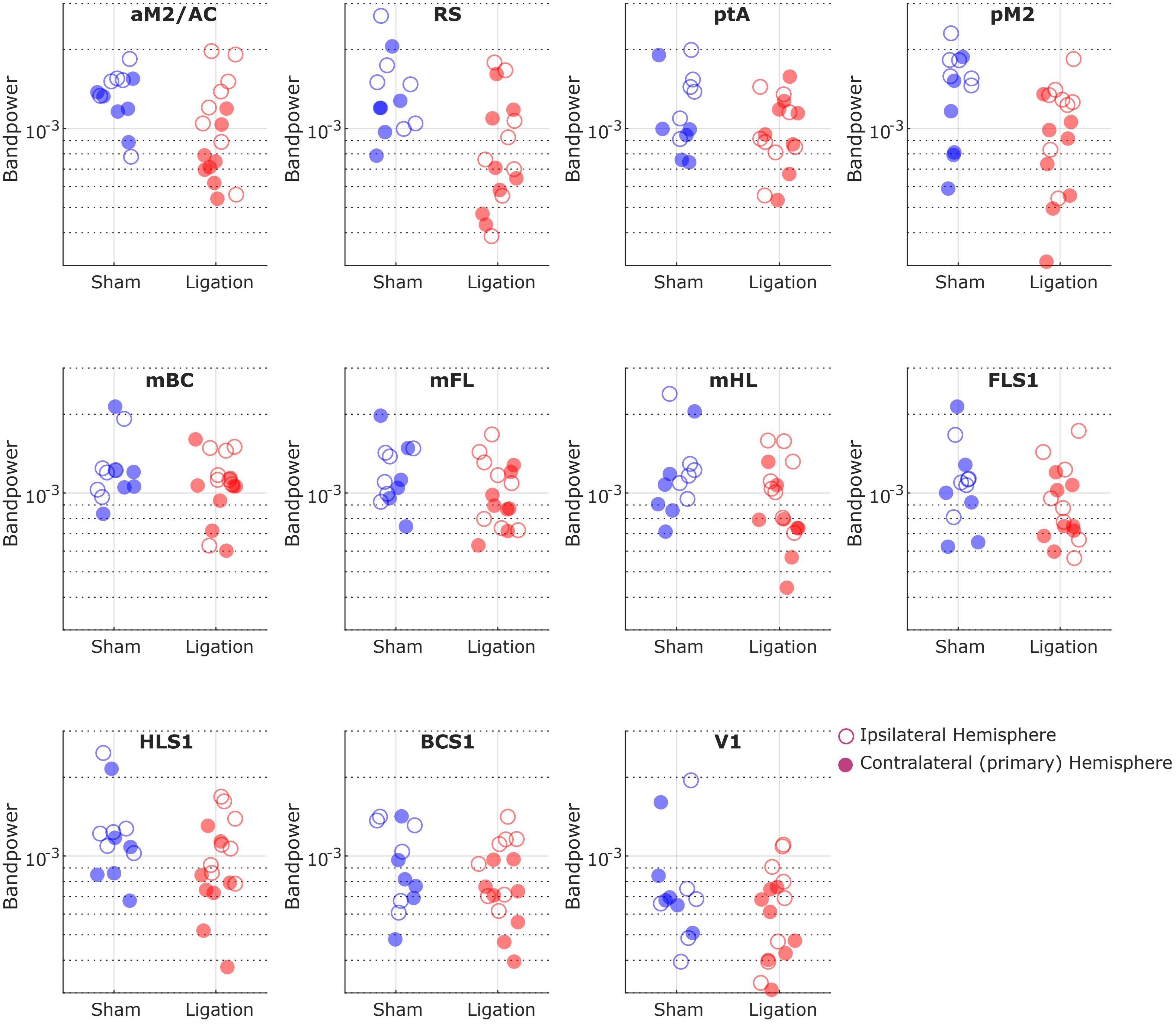

Supplementary Figure 6. Ashby et al., Peripheral Nerve Ligation Elicits Widespread Alterations in Cortical Sensory Evoked and Spontaneous Activity.

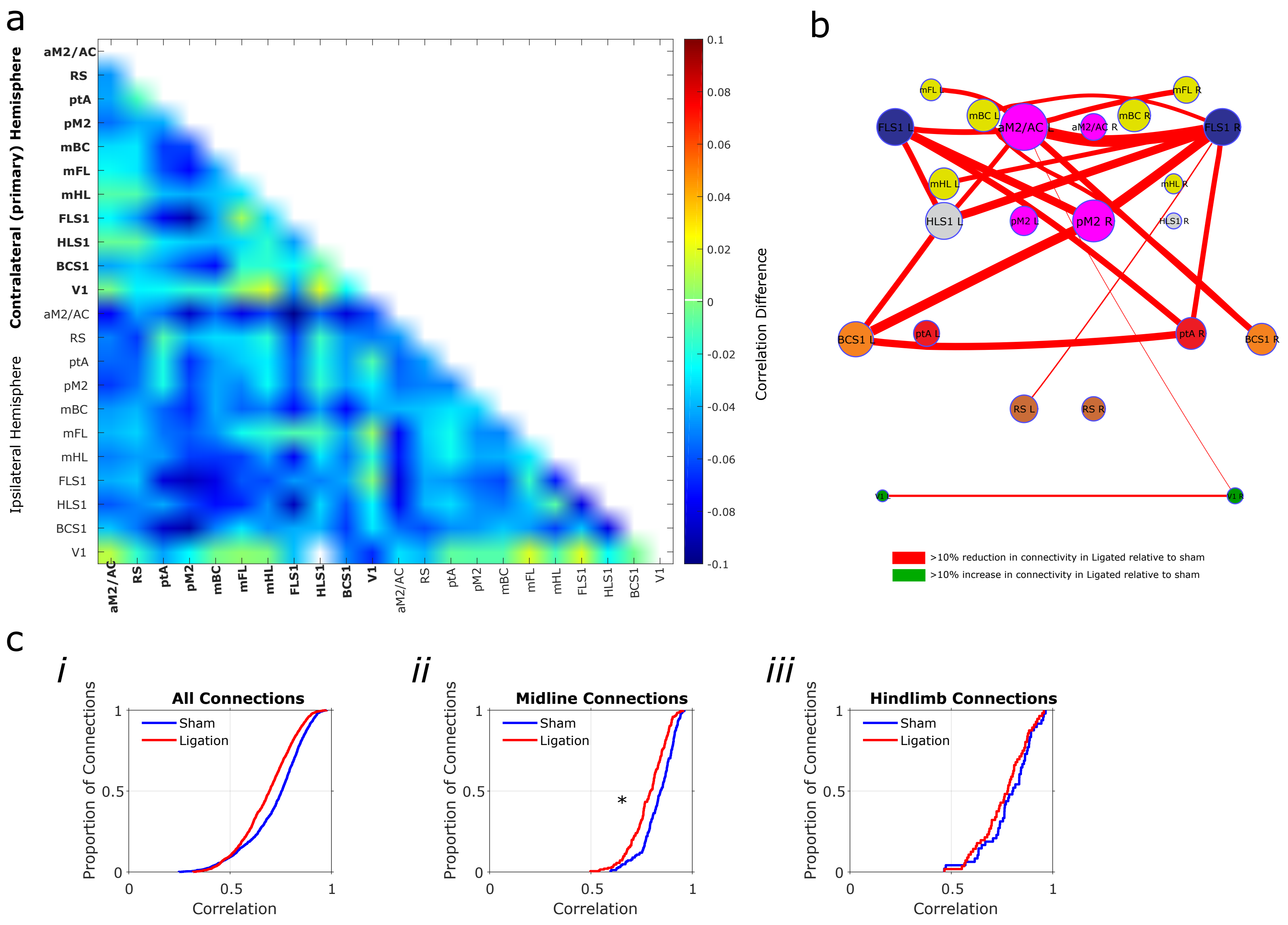

Supplement: Supplementary file 1 — Supplementary Figures [file 41598_2019_51811_MOESM1_ESM.pdf]
